# Uncovering a feedback loop in glioblastoma that reinforces stemness and immunosuppression

**DOI:** 10.1172/JCI205841

**Published:** 2026-05-15

**Authors:** Petros Basakis, Ling-kai Shih, Jiabo Li, Daniel J. Brat

**Affiliations:** 1 Department of Pathology, Northwestern Medicine Malnati Brain Tumor Institute of the Robert H. Lurie Comprehensive Cancer Center, and; 2 Northwestern University Interdepartmental Neuroscience Program, Northwestern University Feinberg School of Medicine, Chicago, Illinois, USA.

## Abstract

Glioma stem cells (GSCs) are a small subset of self-renewing, plastic, and multipotent neoplastic cells in glioblastoma (GBM) that sit at the apex of a cellular differentiation hierarchy. Elucidating pathways that enhance GSC properties and determine their cell-specific interactions within the immunosuppressive GBM microenvironment are critical for developing effective therapeutic approaches. The CLOCK-BMAL1 complex, which is well known for its activity as a circadian rhythm–regulating transcription factor, plays a critical role in maintaining GSC stemness, and the gene encoding CLOCK was found to be amplified in about 5% of GBM cases. Here, Zhou et al. have uncovered a “symbiotic exclusivity” relationship between CLOCK-BMAL1 and TFPI2, which is also amplified in a small proportion of GBM cases. This relationship forms a HIF-1α/NF-κB P65–mediated positive feedback loop that boosts the proliferative and tumor-enhancing capacities of GSC and immunosuppressive microglia. This self-amplifying regulatory circuit represents an opportunity for intervention to inhibit GBM growth.

Glioblastoma (GBM; IDH-WT, WHO grade 4) is the most frequent and malignant primary brain tumor of adults, with median survival ranges of less than 2 years and a 90% relapse rate following standard-of-care therapy (1). Although molecular mechanisms underlying GBM recurrence remain unclear, stem-like cell populations in the tumor microenvironment (TME) are considered one of the main contributors to therapy resistance (2). Glioma stem cells (GSCs) — a small subset of undifferentiated and multipotent GBM cells — retain their aggressive tumorigenic capacity by faithfully reproducing themselves and generating differentiated progeny of neural-like lineage through asymmetric cell division (3). In this issue of the JCI, Zhou et al. provided evidence for a positive feedback signaling loop in GBM that improves our understanding of stemness mechanisms during disease progression (4). A key conceptual advance is the notion of “symbiotic exclusivity,” describing nonoverlapping genomic alterations that are components of a functional regulatory circuit promoting stemness, microglial immunosuppression, and glioma progression.

## The CLOCK-TFPI2 loop depends on HIF-1α/NF-κB P65 signaling

**Figure 1:**
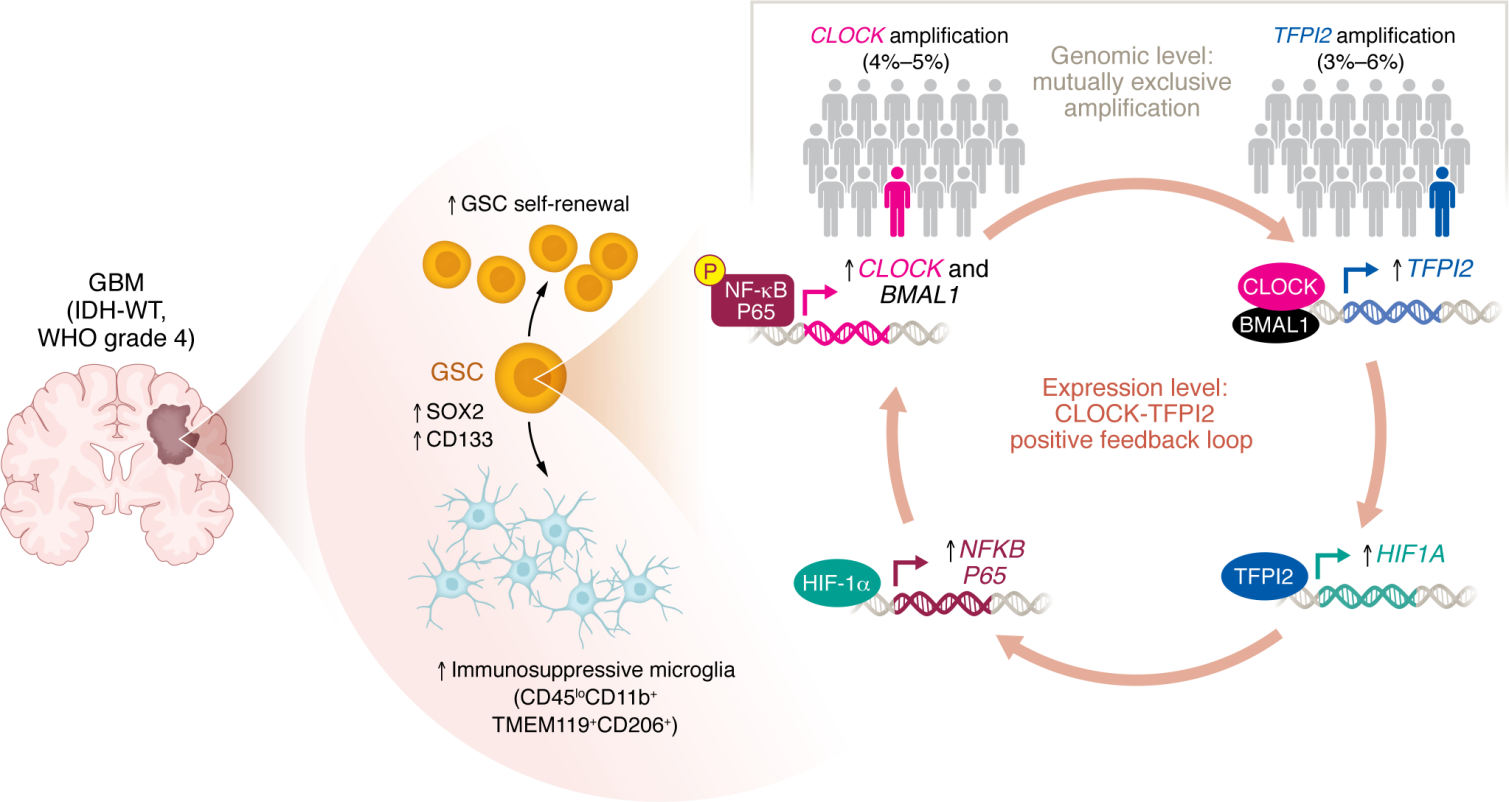
The CLOCK-TFPI2 positive feedback loop compensates for their mutually exclusive amplification. Zhou et al. (4) showed that the self-reinforcing CLOCK-TFPI2 circuit renders the amplification of one gene (CLOCK or TFPI2) sufficient to drive the expression of both and sustain cell-specific mechanisms of GBM progression through the HIF-1α/NF-κB P65 axis. Mechanistically, CLOCK-BMAL1 upregulates TFPI2 in GSC, and in turn, TFPI2-directed HIF1α/NF-κB P65 signaling activation upregulates CLOCK-BMAL1, promoting the proliferation of key GBM-enhancing cell populations, including GSC and immunosuppressive microglia.

The bulk of the experiments performed in this manuscript lead to the careful uncovering of a positive feedback loop in which the CLOCK-BMAL1 complex transcriptionally activates *TFPI2* in GSCs, while TFPI2 reinforces CLOCK signaling through a HIF-1α/NF-κB P65–dependent pathway, thereby sustaining GSC stemness and immunosuppression (Figure 1). Both *CLOCK* and *TFPI2* expression have been linked to GSC stemness, and amplification of either gene is reported in approximately 5% of GBM cases. Building upon this background, Zhou et al. observed a positive correlation between *CLOCK* and *BMAL1* mRNA expression with *TFPI2* mRNA expression in a variety of cellular GBM models, suggesting that such a possible interdependence may exist (4). Subsequent knockdown and overexpression experiments in mouse and patient-derived models of GBM strengthened the evidence for an interdependent relationship and demonstrated that the CLOCK-BMAL1 complex serves as a sufficient regulator of TFPI2 expression in GSCs, and vice versa.

To isolate the key regulators in the proposed pathway, gene set enrichment of hallmark signaling pathways between *shTFPI2* and control samples were compared and suggested that hypoxic and NF-κB signaling pathways were most enriched in the latter, indicating that TFPI2 might direct these networks. These computational findings were confirmed experimentally by overexpressing or knocking down *TFPI2* and demonstrating the hypothesized upregulation or reduction of HIF-1α, P65, and phosphorylated P65 expression. Importantly, P65 was plausibly shown to be a downstream target of HIF-1α, since TFPI2 overexpression led to upregulation of HIF-1α and P65, whereas inhibiting P65 did not directly alter HIF-1α expression. To link TFPI2-induced P65 upregulation back to CLOCK-BMAL1 and complete the loop, experiments were performed to convincingly demonstrate a pattern of enhanced P65 binding on the *CLOCK* and *BMAL1* promoter. The precise mechanism by which TFPI2 causes HIF-1α upregulation was not demonstrated in these studies and could be the subject of future investigation. Finally, pharmacologic inhibition of either HIF-1α or P65 was sufficient to reverse CLOCK-BMAL1 upregulation due to TFPI2 overexpression in a dose-dependent manner.

## CLOCK-BMAL1 and TFPI2: Context-dependent regulators of cancer

The self-reinforcing interplay between CLOCK-BMAL1 and TFPI2 through activation of the HIF-1α/NF-κB P65 intermediate pathway extends our knowledge of how CLOCK-BMAL1 and TFPI2 regulate cancer stem cell (CSC) self renewal. TFPI2 regulates other biologically relevant pathways that support stemness in GBM through the JNK/STAT3 cascade (5). TFPI2 is also an upstream activator of the NF-κB pathway in liver sinusoidal endothelial cells (6), but a downstream target gene of the NF-κB pathway in macrophages, suggesting that the wiring of signaling mediators in TFPI2-related pathways may be cell specific. Interestingly, the role of TFPI2 as a tumor enhancer in GBM also appears to be context dependent, since it acts as a tumor suppressor in lung (7) and pancreatic (8) cancers. The ability of TFPI2 to promote tumor growth may depend on its capacity to exert its procoagulant effects by inhibiting the fibrinolytic system.

On the other hand, and potentially also related to the influence of TFPI2, CLOCK-BMAL1 promotes tumor-supportive signaling via the OLFML3/HIF-1α axis in GSC (9). Like TFPI2, CLOCK-BMAL1 has also been shown to have tumor-suppressive functions in other forms of cancer; in colorectal carcinomas, BMAL1 suppresses YAP1-dependent Hippo pathway activity while stimulating Wnt signaling to attenuate CSC self renewal (10). In metastatic lung cancer, BMAL1 suppresses invasion through the PI3K/Akt/MMP-2 pathway in a p53-independent manner (11). Therefore, the amplification patterns and regulatory pathways directed by CLOCK and TFPI2 in GBM, as demonstrated in the current study, may not be generalizable to other cancers.

## The self-reinforcing CLOCK-TFPI2 loop: a symbiotic exclusivity case

Mutually exclusive genomic amplifications are often interpreted as indicators of functional redundancy within the same oncogenic pathway. The identification of a feedback loop between CLOCK and TFPI2 provides a mechanistic explanation for this pattern, suggesting that amplification of either regulator may be sufficient to sustain the signaling circuit that promotes glioma stemness and immunosuppression. Zhou et al.’s analysis of the Cancer Genome Atlas Glioblastoma (TCGA-GBM) data showed that *CLOCK* and *TFPI2* amplification were virtually mutually exclusive, with the 4%–5% of samples with *CLOCK* amplification showing minimal overlap with the 3%–6% of samples with *TFPI2* amplification (4). The lack of overlap between *CLOCK* and *TFPI2* amplification at the genomic level might suggest that their coenrichment could be a fitness disadvantage as long as there is a mechanism by which a single amplification (or another pathway initiator) is a sufficient driver. The uncovered positive feedback loop between CLOCK-BMAL1 and TFPI2 provides an explanation for their dependency and need for only one stimulatory event. Indeed, dual *shRNA*-directed knockdown of *CLOCK* and *TFPI2* did not offer any additive benefit in reducing GSC self renewal or shortening survival compared with single knockdown, strengthening the claim of mutual dependency. Although their coamplification is biologically redundant, the presence of both arms of the regulatory pathway is needed for the positive feedback loop to drive stemness-linked tumor progression.

Aside from *CLOCK* and *TFPI2* gene amplification, which were infrequently noted in GBM samples, other initiators or enhancers might be expected. For example, hypoxic upregulation of HIF-1α might lead to enhanced signaling through this pathway and potentially explain the enrichment of GSC in hypoxic, perinecrotic zones of GBM (12, 13). Overall, Zhou et al.’s results indicate that the mutual exclusivity at the genomic level reflects a symbiotic exclusivity pattern at the intracellular signaling level: the symbiosis between CLOCK-BMAL1 and TFPI2 in a positive feedback loop leads to complete compensation for their genomic mutual exclusivity and for pathway activation in the absence of either amplification event.

## Immunosuppressive microglia: exclusive target of the CLOCK-TFPI2 loop

Thirty-to-fifty percent of all cell types in GBM are tumor-associated macrophages/microglia (TAMs). These include activated, brain-resident microglia and bone-marrow–derived macrophages (BMDMs), representing the largest nonneoplastic population in GBM and a plausible therapeutic target (13). The role of tumor-associated microglia in the GBM TME is often underappreciated, especially when compared with BMDMs. In necrotic GBMs, BMDMs dominate, representing 80% of TAMs, with the highest density in the perinecrotic niche, while the much smaller subset of microglia is found mostly at the leading infiltrative edge (13, 14). As the disease advances, the dominant phenotypic profiling of TAMs transitions from proinflammatory microglia to immunosuppressive and protumorigenic BMDMs (15).

Despite these prevalent patterns, in the present study, immunosuppressive microglia (CD45^low^CD11b^+^TMEM119^+^CD206^+^) were the only significantly reduced immune cell population following dual pharmacologic inhibition of pathways downstream of CLOCK-BMAL1 and TFPI2 in tumor-bearing, immunocompetent mice. Cell-specific pathway activation may explain why immunosuppressive microglia — and not BMDMs — are the TAMs affected through indirect targeting of both modulators. GSC-secreted TFPI2 serves as a selective ligand for the CD51 receptor on microglia (5). Activated CD51 polarizes microglia through STAT6 signaling into an immunosuppressive state. CLOCK-BMAL1 promotes their infiltration in the immunosuppressive TME through activation of the CLOCK/OLFML3/HIF1α/LGMN/CD162 axis (9). Another possibility for the selective targeting of immunosuppressive microglia is their exclusive polarization through PI3K/AKT/mTOR signaling (16). CLOCK-driven phosphorylation of AKT and mTOR in GSCs may signal mTOR activation in microglia to induce their immunosuppressive phenotype. Thus, the CLOCK–TFPI2 loop appears to regulate immunosuppressive polarization of microglia more than systemic monocyte recruitment.

## Therapeutic targeting of the CLOCK-TFPI2 loop

Successful therapeutic interventions targeted at the CLOCK-TFPI2 loop must consider its multiple arms of activation and downstream effectors. Since direct pharmacological inhibitors of CLOCK and TFPI2 are not available, Zhou et al. attempted inhibition of their downstream targets using Stiripentol, which blocks the HIF-1α target LDHA, together with the JNK inhibitor JNK-IN-8 or STAT3 inhibitor WP1066 (4). Simultaneous inhibition of CLOCK and TFPI2 downstream pathways in tumor-bearing mice showed modest effects but was insufficient to cause complete tumor regression. However, when this strategy of using Stiripentol and JNK-IN-8 or WP1066 was combined with anti-PD1 therapy, which maintains relatively high T cell activity in GBM’s T cell–exhausting TME, the percentage of tumor-free mice was doubled (25% to 50%–62.5%) (5, 9). Similar simultaneous targeting of CLOCK, TFPI2, and PD1 in recurrent GBM mice might bolster the significance of the findings, given the biological relevance of the CLOCK-TFPI2 interplay to stemness — one of the primary mechanisms thought to drive disease relapse. Until direct inhibition of CLOCK and TFPI2 becomes therapeutically feasible, future trials could consider the integration of inhibitors of HIF-1α (e.g., acriflavine), NF-κB P65 (e.g., SC75741) and mTOR (e.g., rapamycin) signaling, all of which can suppress GBM cell populations, including GSCs and immunosuppressive microglia.

## Conclusion

The mutually exclusive amplification of *CLOCK* and *TFPI2* in GBMs suggested that a dependency between these two regulators may exist, which was demonstrated by the uncovering of a self-reinforcing feedback loop. The authors successfully demonstrated that TFPI2-directed upregulation of the HIF-1α/NF-κB P65 signaling cascade supports the symbiotic role of CLOCK-BMAL1 and TFPI2 as tumor enhancers in GBM. Additional validating studies will be needed, given the complex, dynamic, and cell-specific changes of the GBM TME across various stages of the disease. Nevertheless, this study convincingly demonstrates that a novel CLOCK-TFPI2 symbiotic exclusivity pattern exists in a positive feedback loop and is sufficient to propagate stem-like and immunosuppressive cell populations, propelling GBM progression.

## Conflict of interest

The authors have declared that no conflict of interest exists.

## Funding support

This work is the result of NIH funding, in whole or in part, and is subject to the NIH Public Access Policy. Through acceptance of this federal funding, the NIH has been given a right to make the work publicly available in PubMed Central.

- The National Institutes of Health/National Cancer Institute under awards R01CA247905, R01CA295560, and P50CA221747.
- The Department of Defense under award 14301880.
- The Dixon Foundation.

## Version 1 05/15/2026

Electronic publication
